# Analytical discrepancy in platelet counting in *MYH9*–related disease: a case report

**DOI:** 10.1093/labmed/lmag078

**Published:** 2026-10-15

**Authors:** Marco De Girolamo, Anna Marrocchi, Francesca Vitone, Margherita Scapaticci, Monica Bovara, Tommaso Paolo Papillo, Rita Mancini

**Affiliations:** Alma Mater Studiorum, Università di Bologna, Bologna, Italy; LUM, AUSL di Bologna, Bologna, Italy; LUM, AUSL di Bologna, Bologna, Italy; LUM, AUSL di Bologna, Bologna, Italy; LUM, AUSL di Bologna, Bologna, Italy; LUM, AUSL di Bologna, Bologna, Italy; LUM, AUSL di Bologna, Bologna, Italy

**Keywords:** *MYH9*–related disease, thrombocytopenia, giant platelets, Döhle-like bodies, giant platelets, fluorescence counting, hematology

## Abstract

**Introduction:**

*MYH9*–related disease (*MYH9*-RD) is a rare autosomal dominant disorder characterized by congenital macrothrombocytopenia, with giant platelets and variable extrahematologic manifestations. It is frequently underdiagnosed and misclassified as immune thrombocytopenia.

**Methods:**

A 31-year-old pregnant woman undergoing routine first-trimester testing was shown to have severe thrombocytopenia (platelet count = 5.000 × 10³/µL) by impedance counting.

**Results:**

Platelet histograms and scattergrams generated on a Sysmex XN-10 hematology analyzer suggested giant platelets, while fluorescence counting using a Sysmex XN-20 hematology analyzer yielded a higher platelet count (19.000 × 10³/µL), considered a more plausible estimate in the presence of giant platelets. Peripheral blood smear examination excluded platelet clumping and demonstrated numerous giant platelets and basophilic neutrophilic inclusions, subsequently interpreted as Döhle-like bodies. Further medical history revealed a previous diagnosis of *MYH9*-RD. Longitudinal evaluation showed persistently low but stable platelet counts, no kidney involvement, and mild liver enzyme elevation.

**Discussion:**

This case highlights the diagnostic value of integrating automated hematology data with peripheral blood smear examination in inherited macrothrombocytopenias. Platelet counts in the presence of giant platelets may be underestimated by impedance-based analyzers because of size-dependent misclassification, whereas fluorescence-based counting may provide a more reliable estimate. Recognition of characteristic morphologic features, including Döhle-like bodies, is essential for identifying *MYH9*-RD and distinguishing it from immune thrombocytopenia, thereby avoiding inappropriate diagnostic and therapeutic interventions.

## Introduction

*MYH9*–related disease (*MYH9*-RD) is a rare autosomal dominant disorder characterized by a broad spectrum of hematologic and extrahematologic manifestations.^1^ It is caused by pathogenic variants in the *MYH9* gene, which encodes nonmuscle myosin heavy chain IIA (NMMHC-IIA), a cytoskeletal protein involved in several essential cellular processes, including cytokinesis, cell polarization, and migration.^2^

The disease is present from birth and is characterized by congenital thrombocytopenia associated with macrothrombocytes and giant platelets on peripheral blood smear.^3^ A characteristic feature of the disease is the presence of cytoplasmic inclusions within neutrophils, corresponding to aggregates of mutant NMMHC-IIA, which appear as Döhle-like bodies and can be detected by immunofluorescence staining.^4^ The severity of thrombocytopenia ranges from mild to severe but generally remains stable throughout life. Although some patients remain asymptomatic, a bleeding tendency is the main clinical manifestation and is usually proportional to the degree of thrombocytopenia. Beyond the hematologic phenotype, *MYH9*-RD may be associated with extrahematologic manifestations, including sensorineural hearing loss, glomerular nephropathy, presenile cataracts, and elevated liver enzymes.^5^

The diagnosis can be suspected on the basis of characteristic clinical, laboratory, and morphologic findings, particularly macrothrombocytopenia and inclusion of Döhle-like bodies in neutrophils, but it is confirmed by molecular genetic testing demonstrating a heterozygous pathogenic variant in the *MYH9* gene.^1^ The differential diagnosis includes both inherited and acquired thrombocytopenias, particularly immune thrombocytopenia, with which *MYH9*-RD may be confused, potentially leading to inappropriate therapeutic interventions.

We report a case of previously diagnosed *MYH9*-RD in a pregnant woman with severe macrothrombocytopenia, illustrating the importance of recognizing inherited thrombocytopenia and distinguishing it from acquired causes. The marked discrepancy between impedance-based and fluorescence-based platelet counts represented an important laboratory clue to the presence of giant platelets.

The study was conducted in accordance with the principles of the Declaration of Helsinki. Written informed consent for publication of the patient’s clinical data and accompanying images was obtained.

## Methods

A 31-year-old pregnant woman underwent routine laboratory testing during the first trimester of pregnancy. Peripheral venous blood samples were collected in EDTA-anticoagulated tubes according to standard laboratory procedures. A complete blood cell count analysis was initially performed using a Sysmex XN-10 automated hematology analyzer; the results revealed severe thrombocytopenia, with a platelet count of 5 × 10³/µL measured by the impedance method; all other hematologic parameters were within the reference ranges, including red blood cell count (5 × 10¹²/L), hemoglobin (13.1 g/dL), hematocrit (41.1%), mean corpuscular volume (82 fL), and white blood cell count (5.06 × 10⁹/L).

## Results

The platelet histogram (Figure 1A) showed an abnormal distribution of platelets extending toward larger platelet volumes, suggesting the presence of giant platelets. In addition, the WNR and WDF leukocyte scattergrams demonstrated a distinct population of low-fluorescence events, appearing as a blue cloud consistent with giant platelets (Figure 1B, C). Given the clinical presence of severe thrombocytopenia, platelet counting was repeated using the fluorescence platelet counting method (PLT-F) on a Sysmex XN-20 automated hematology analyzer. This process yielded a platelet count of 19 × 10³/µL, markedly higher than that obtained by the impedance method (Figure 1D). No manual platelet count or reference flow cytometric platelet count was performed. Therefore, although the PLT-F result was considered a more plausible estimate than the impedance-based count in the presence of giant platelets, the true platelet count could not be definitively established.

**Figure 1:**
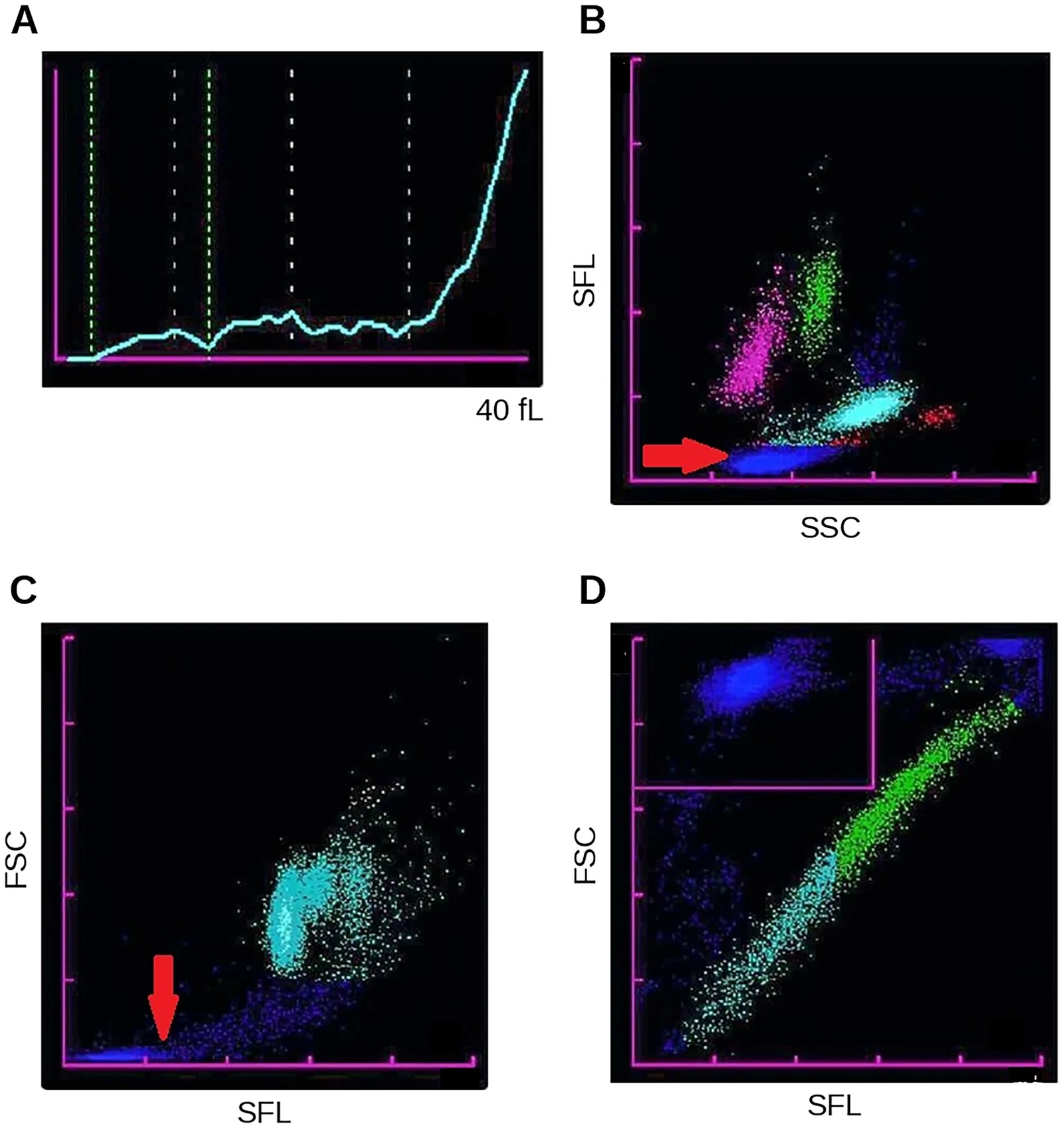
Automated hematology analyzer findings in severe macrothrombocytopenia. **(A)** Platelet histogram obtained by impedance-based platelet counting shows an abnormal distribution extending toward larger platelet volumes, consistent with the presence of giant platelets. The platelet count by impedance was 5 × 10³/µL. **(B)** WDF and **(C)** WNR scattergrams show a distinct population of low-fluorescence events (indicated by arrows), consistent with giant platelets. **(D)** The fluorescence-based platelet counting scattergram shows a clear platelet cluster in the immature platelet fraction area (indicated in green) consisting of giant platelets. FSC indicates forward scatter; SFL, side fluorescence light; SSC, side scatter.

Given the presence of severe thrombocytopenia and the patient’s pregnancy, a peripheral blood smear was prepared for morphologic confirmation. Blood smear morphology was initially assessed using an automated digital imaging system (Sysmex/CellaVision DI-60) and subsequently reviewed by conventional light microscopy. Smears were stained with May-Grünwald Giemsa stain and examined for platelet morphology and leukocyte cytoplasmic inclusions. The CellaVision system did not automatically identify or classify neutrophils as containing Döhle-like bodies; the morphologic findings were identified during operator review of the digital images and subsequently confirmed by conventional light microscopy. Platelet clumping was excluded, confirming true thrombocytopenia. Numerous giant platelets were observed, several of which were comparable in size to erythrocytes (Figure 2A). In addition, basophilic cytoplasmic inclusions located at the periphery of neutrophil cytoplasm were identified (Figure 2B). Based on these findings, the patient was contacted for further clinical history; she reported a previous diagnosis of *MYH9*-RD. In the context of the known diagnosis, the neutrophil inclusions were interpreted as the characteristic Döhle-like bodies associated with *MYH9*-RD.

**Figure 2:**
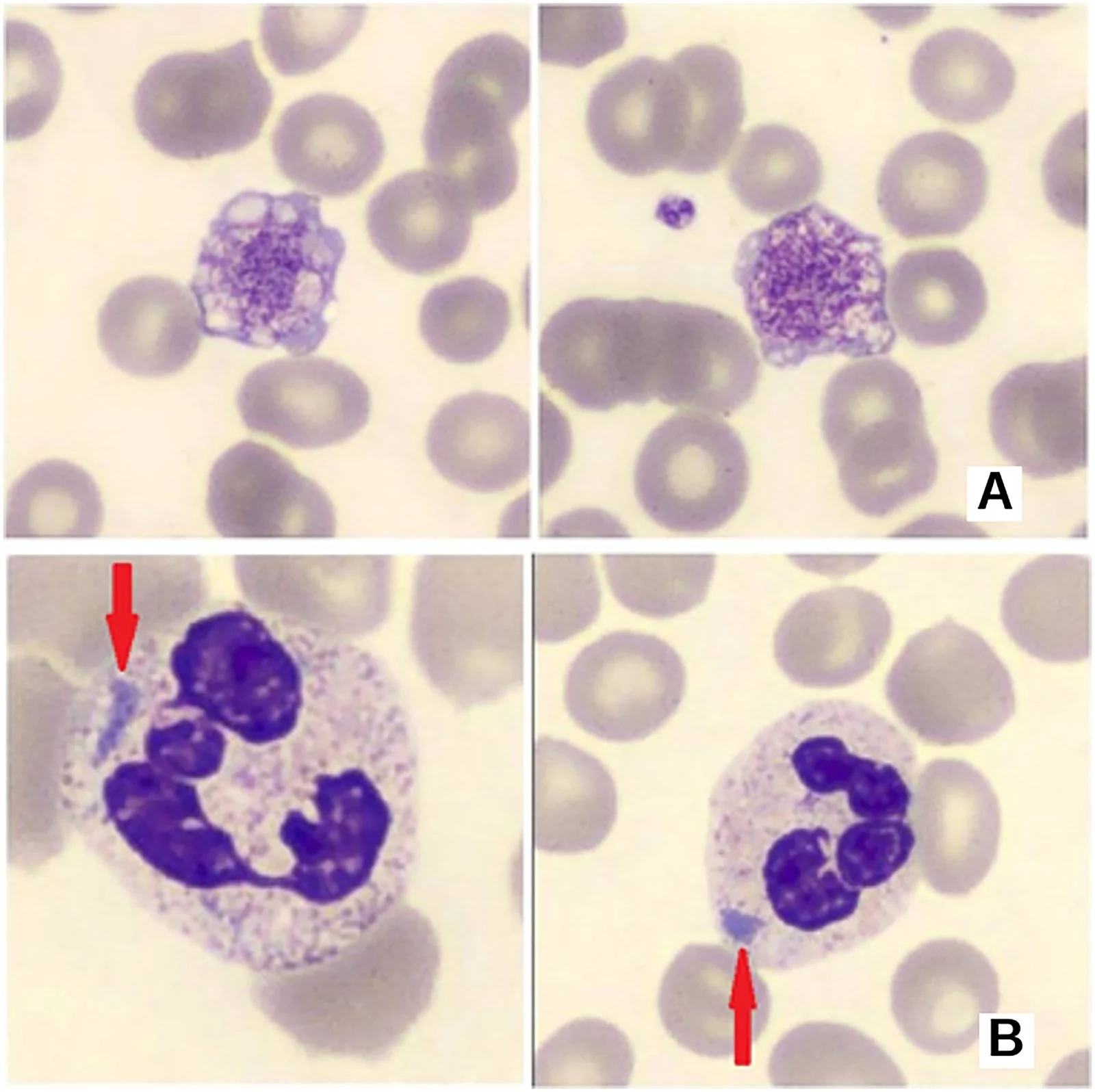
Peripheral blood smear findings in *MYH9*–related disease (May-Grünwald Giemsa). **(A)** Representative giant platelets, some approaching the size of erythrocytes, consistent with marked macrothrombocytopenia (original magnification, x1000). **(B)** Neutrophil showing a basophilic cytoplasmic inclusion located at the periphery of the cytoplasm (arrow), interpreted as a Döhle-like body in the context of the patient’s known *MYH9*–related disease (original magnification, x1000).

## Discussion

The main educational value of this case lies in the recognition of marked macrothrombocytopenia and the importance of distinguishing inherited thrombocytopenia from acquired forms, particularly immune thrombocytopenia. In this setting, the marked discrepancy between platelet counts obtained using impedance-based and fluorescence-based methods represented an important analytical clue to the presence of giant platelets, although this finding is not specific to *MYH9*-RD. This discrepancy can be explained by the presence of giant platelets, which may exceed the size threshold used by impedance-based analyzers and consequently be misclassified as erythrocytes, leading to substantial underestimation of the platelet count.^6^ In contrast, fluorescence-based platelet counting identifies platelets by combining fluorescent labelling of platelet-specific intracellular components with forward light scatter, allowing more accurate discrimination of large platelets from erythrocytes and other particles.^7^ In the present case, the fluorescence-based platelet count (19 × 10³/µL) was considered a more plausible estimate than the impedance-based count (5 × 10³/µL) given the marked macrothrombocytopenia demonstrated on peripheral blood smear examination. However, no manual platelet count or reference flow cytometric platelet count was available for comparison. Therefore, although fluorescence-based counting provides improved discrimination of giant platelets compared with impedance-based methods, the PLT-F value cannot be regarded as a definitive measure of the true platelet count.^7^

The extremely low impedance-based platelet count required analytical verification using an alternative platelet-counting method and peripheral blood smear examination. The abnormal platelet histograms and the low-fluorescence population observed on the WNR and WDF scattergrams provided additional analytical clues to the presence of giant platelets and contributed to the interpretation of the thrombocytopenia. Peripheral blood smear examination demonstrated numerous giant platelets and Döhle-like bodies, the characteristic morphologic features of *MYH9*-RD. Longitudinal follow-up showed persistent thrombocytopenia, with platelet counts ranging from 19 × 10³/μL to 45 × 10³/μL, consistent with the chronic nature of the disorder.

Evaluation of extrahematologic manifestations showed preserved kidney function, with normal serum creatinine, estimated glomerular filtration rate, and urinalysis findings, making clinically significant kidney involvement unlikely at the time of assessment. Mild elevation of aminotransferase levels was observed, suggesting possible hepatic involvement, which has been described in *MYH9*-RD, although alternative causes could not be excluded.^2^

This case highlights the importance of recognizing marked macrothrombocytopenia as a potential clue to inherited thrombocytopenia, particularly in patients who might otherwise be considered to have immune thrombocytopenia. Integration of automated analyzer data with careful peripheral blood smear examination is essential for identifying characteristic morphologic features and avoiding inappropriate diagnostic and therapeutic interventions.

## Data Availability

The data supporting the findings of this study are available from the corresponding author upon reasonable request.
